# Supplementary material for: Resolving the full spectrum of human genome variation using Linked-Reads
Source: Genome Res. 2019 Apr;29(4):635–45. doi: 10.1101/gr.234443.118 (PMC6442396; doi:10.1101/gr.234443.118)
Supplement: Supplemental Material [file supp_gr.234443.118_Supplemental_longranger-2.2.zip › longranger-2.2.tar.bz2!/lr2.2-public/lib/stan/cmdstan/stan/doc/api/html/classstan_1_1math_1_1_l_d_l_t__factor.html]

Stan: stan::math::LDLT\_factor< T, R, C > Class Template Reference


|  |  |
| --- | --- |
|  | Stan  2.5.0  probability, sampling & optimization |


- Main Page
- Related Pages
- Namespaces
- Classes
- Files

- Class List
- Class Index
- Class Hierarchy
- Class Members

All Classes Namespaces Files Functions Variables Typedefs Enumerations Enumerator Friends Macros Pages

- stan
- math
- LDLT\_factor

stan::math::LDLT\_factor< T, R, C > Class Template Reference

`#include <LDLT_factor.hpp>`

## Detailed Description

### template<typename T, int R, int C> class stan::math::LDLT\_factor< T, R, C >

Definition at line 16 of file LDLT\_factor.hpp.

---

The documentation for this class was generated from the following file:

- src/stan/math/matrix/LDLT\_factor.hpp


---

[ Stan Home Page ]

*© 2011–2014,
Stan Development Team.*
